# Biology—The Path Ahead

**DOI:** 10.3390/biology1010001

**Published:** 2011-09-01

**Authors:** Christopher A. O’Callaghan

**Affiliations:** Henry Wellcome Building for Molecular Physiology, University of Oxford/ Roosevelt Drive, Oxford, OX3 7BN, UK; E-Mail: chris.ocallaghan@ndm.ox.ac.uk; Tel.: +44-1865-287789; Fax: +44-1865-287787

## Biology—Exciting Times

There has never been a more exciting time to study the science of living systems. Contemporary biology is a vibrant field which grows stronger year on year. Biological scientists have access to powerful new tools and techniques that even recently would have seemed like science fiction. We are enjoying ‘a wellspring of technical advancements’ [1]. The consequence is a deeper and wider understanding of living systems. Older approaches remain important and often essential, but new unbiased and often fast approaches are helping us to deploy our traditional approaches more rationally. The result of this synergy of old and new is an explosion in the field of biological sciences. It may seem daunting to consider what lies ahead and the challenges will be great, but the pace of discovery is increasingly rapid. 

The revolutions taking place result in part from technological advances in our ability to undertake ever larger-sized analyses at all scales from the sub-atomic to the global. Thus, for example, more and more genomes are being sequenced and compared, large global scale datasets are being analyzed and more and bigger biological structures are being solved. In almost every area of biological investigation larger datasets are available; this can be from wider, often unbiased, data harvesting, such as genome sequencing, or from highly focused, data-rich studies, such as real-time neurophysiological monitoring. 

The huge advances in biology are far from sterile. They are already influencing the present and will continue to shape the future in diverse areas such as medicine, food production, synthetic biology and environmental care. Enormous effort is being invested into translating these advances for global benefit and it is difficult to overestimate the impact that the study of biology has already had and will continue to have. This translational work is biology too and rightly so. 

**Figure 1:**
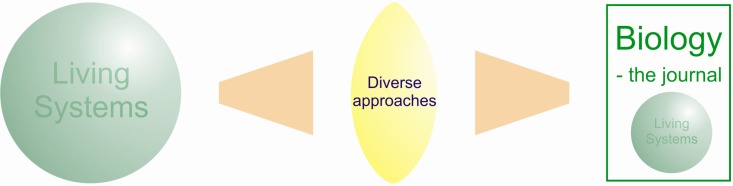
*Biology* will be an inclusive journal. Living systems are studied through the lenses of many diverse experimental and theoretical approaches and this journal will feature insight from this wide range of approaches.

## *Biology*—the Journal

Biology, the subject, is at the forefront of scientific progress and this is where the journal *Biology* will be (Figure 1). Biological systems are typically complex and inter-related; understanding this complexity is a great challenge. However, a major strength of biological science is the diversity of approaches that biological scientists apply to their research problems. A biological system may be investigated in many different ways using genetic approaches, structural biology, comparative biology, population biology and so on. This diversity keeps the field dynamic and flexible, so driving forward progress. 

*Biology* is a new journal and its launch offers an exciting possibility to create a journal that reflects this diversity within biological science. The journal will bring together studies employing the varied experimental and theoretical approaches that are fuelling biological discovery. Biology will be an inclusive journal that embraces all these disciplines and sub-disciplines. With the explosion of biological discovery, there is an explosion in publication. The rate of discovery in most scientific groups is accelerating and there is a pressing need to simplify and accelerate the process of publication. *Biology*, the journal, will do this. It will be a fully peer-reviewed publication with a rapid and economical route to open access publication.

## *Biology*—the Scope of the Journal

We regard biology as the study of any biological system at any scale from the global to the sub-atomic. Biology encompasses the diversity that constitutes the living world in its myriad manifestations. We make no distinction between biology, biochemistry, genetics, immunology, paleontology, zoology, biophysics, neuroscience, developmental biology, mathematical biology, bacteriology, systems biology, virology, botany, genomics, medicine, parasitology, pharmacology, proteomics or any other biological disciplines. They are all part of the same subject, the study of life.

Both the subject and the approaches to its study are exciting and diverse. Bringing all these studies together in a multi-disciplinary manner should drive the field forward by providing an overview that may not be possible from studies conducted only at a particular scale or using only one modality of investigation.

Biologists study amazing and complex systems using an incredibly wide repertoire of approaches. *Biology*, the journal, will capture this diversity and publish across the spectrum of the field. As well as standard research articles we will also publish reviews, commentaries, hypotheses and descriptions of new methods.

## Why Publish in *Biology*?

*Biology* will provide very rapid dissemination of robust scientific findings. All articles will be peer-reviewed, but the focus will be on determining that the work is scientifically sound rather than trying to predict its future impact. Good results will stand the test of time and the priority for scientists now is to disseminate their findings.

Writing in 1974, Francis Crick, reflecting on the impact of the publication of the work on the structure of DNA that won him the Nobel Prize, suggested that it would be for historians to decide the impact of this work [2]. He noted wisely that ‘The genetic code was not revealed all in one go but it did not lack for impact once it had been pieced together.’ Much biological work is like the elucidation of the genetic code. Even if each individual report does not have a huge impact, the totality of these reports creates a new picture of the world we inhabit and has ongoing impact. 

The editorial panel and the referees of the journal *Biology* will not waste your time or their time trying to assess the impact of your article. We will assess whether it is scientifically sound and properly written. If work is well conducted and properly documented, then it is necessarily a contribution to the field and only the future can determine its true impact or significance.

We are an open access journal. When most research is funded by tax payers or charitable donors, it seems appropriate that the reports of this research should be freely available to all. However, open access comes with a price tag as researchers have to fund the publications. *Biology* has a streamlined publications process that uses technology to keep the price of publication down. Editors offer their services without payment. The result is that our charges are low and some of the most economical available. Indeed submission is free until 2013. The less money that is spent on publishing, the more there is to spend on discovery.

We want to accelerate the progress from discovery to dissemination. Review and a decision on publication will be rapid. The time that scientists have is precious and is better spent on discovery than on rewriting articles or trying to persuade journals of their potential impact.

The key tests for publication in *Biology* are simple. Firstly, is the work scientifically robust? Secondly, is the paper sensibly argued and properly written? We have no limit to the number of papers that we can publish and there is no minimum or maximum page length for a *Biology* article. If you feel something is ready for dissemination please submit it and we will aim to give you a rapid peer-reviewed decision and fast publication if accepted.

Biology has a superb and diverse editorial team, which includes a Nobel laureate. We are committed to making the journal a success. Our aim is to promote research by reducing the time that publishing results takes out of busy research schedules. The journal will in time be indexed in Pubmed and other standard search and indexing services. It will benefit from the publisher’s experience over more than ten years with a range of successful online journals and the crisp web interface and submission procedure. The launch of a new journal is an exciting moment and we encourage you to submit your articles to *Biology* now.
